# Ultrabroadband and Highly Sensitive Short‐Wave Infrared Molecular Fingerprinting via Acoustic MXene Plasmons

**DOI:** 10.1002/advs.75346

**Published:** 2026-04-16

**Authors:** Changhoon Park, Jisung Kwon, Nu‐Ri Park, Hyerim Kim, Hyeju Kim, Yury Gogotsi, Chong Min Koo, Myung‐Ki Kim

**Affiliations:** ^1^ KU‐KIST Graduate School of Converging Science and Technology Korea University Seoul Republic of Korea; ^2^ A. J. Drexel Nanomaterials Institute and Department of Materials Science and Engineering Drexel University Philadelphia Pennsylvania USA; ^3^ School of Advanced Materials Science & Engineering Sungkyunkwan University Suwon‐si Republic of Korea; ^4^ Department of Materials Science and Engineering Korea University Seoul Republic of Korea; ^5^ School of Chemical Engineering Sungkyunkwan University Suwon‐si Republic of Korea; ^6^ Department of Integrative Energy Engineering College of Engineering Korea University Seoul Republic of Korea

**Keywords:** 2D materials, acoustic plasmons, MXenes, MXene plasmons, surface‐enhanced infrared absorption (SEIRA) spectroscopy

## Abstract

Surface‐enhanced infrared absorption (SEIRA) spectroscopy has emerged as a powerful technique, amplifying inherently weak molecular vibration signatures to enable ultrasensitive detection of molecular structure and dynamics. However, conventional SEIRA platforms based on noble metals or 2D materials are fundamentally constrained by their narrow spectral bandwidths and limited access to high‐frequency vibrational modes. Here, we demonstrate an ultrabroadband SEIRA approach that overcomes these limitations by activating acoustic plasmon modes in two‐dimensional Ti_3_C_2_T*_x_* MXene. These acoustic plasmons provide deep subwavelength confinement, compressing wavelengths by more than two orders of magnitude relative to free space in the short‐wave infrared (SWIR), and sustaining an unprecedented spectral bandwidth of approximately 5000 cm^–^^1^. Using this platform, we achieve simultaneous detection of distinct vibrational fingerprints—from high‐frequency CH_3_ combination bands near 4700 cm^−1^ to low‐frequency out‐of‐plane bending modes around 700 cm^−1^—in ultrathin analytes such as 8 nm PMMA and 10 nm graphene oxide films, with up to an order‐of‐magnitude sensitivity enhancement. These results establish acoustic plasmon modes in MXene as a transformative foundation for ultrabroadband, high‐sensitivity deep‐infrared spectroscopy, paving the way for next‐generation molecular sensing technologies.

## Graphical abstract

Acoustic plasmons in Ti_3_C_2_T_x_ MXene strongly compress infrared electromagnetic fields and enhance light–matter interactions at the nanoscale. This platform couples broadband plasmon excitations with molecular vibrations, enabling simultaneous sensing of high‐ and low‐frequency vibrational modes in ultrathin PMMA and graphene oxide films, and extending SEIRA functionality far beyond conventional narrowband limits.

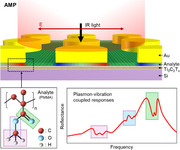

## 1 Introduction

Plasmons are collective oscillations of free electrons that couple strongly with electromagnetic radiation. These excitations have attracted significant attention in nanophotonics because they enable deep subwavelength confinement of optical fields and strong local field enhancement. In particular, plasmons supported by 2D materials can compress the wavelength of infrared (IR) light to the nanoscale, providing an effective platform for enhancing light–matter interactions. Such extreme confinement mitigates the mismatch between molecular dimensions and the wavelength of light, thereby providing substantial signal enhancement for chemical sensing. Among various sensing strategies, surface‐enhanced infrared absorption (SEIRA) spectroscopy has emerged as a powerful approach to amplify inherently weak molecular vibrational signals through localized electromagnetic field enhancement [1, 2, 3, 4]. With their ability to compress wavelengths by more than two orders of magnitude, 2D materials hold great promise for highly sensitive SEIRA‐based molecular fingerprint detection. However, their intrinsically low electron density restricts plasmon dispersion to relatively low frequencies [5, 6]. As a result, most SEIRA devices based on conventional 2D materials such as graphene operate primarily in the mid‐infrared (MIR) regime [1, 2, 3, 4], typically below ∼3300 cm^−1^, limiting their capability to probe high‐frequency vibrational features in the short‐wave infrared (SWIR) range, such as O─H stretching overtones and combination bands [7, 8]. In addition, their strong frequency selectivity often yields narrow spectral bandwidths, hindering broadband molecular profiling within a single device. These limitations underscore the need for new 2D plasmonic materials that combine high sensitivity, broad spectral coverage, and extended dispersion into the SWIR.

MXenes, a family of 2D transition metal carbides, nitrides, and carbonitrides with the general formula M_n+1_X_n_T*_x_*, have recently attracted considerable attention owing to their chemical tunability and versatile optical properties [9]. They show various colors with interband transitions spanning the UV–vis–NIR [10, 11] depending on their chemical composition, metallic responses at low frequencies with applications in EMI shielding [12] and nanophotonics [13, 14, 15], pronounced nonlinear optical responses [16, 17], and tunable characteristics through intercalation or engineering surface terminations [18, 19, 20]. Among them, Ti_3_C_2_T*_x_* MXene [21] is particularly notable for its exceptionally high carrier density [6] and conductivity [9] exceeding 24 000 S/cm, which supports surface plasmon modes extending from the NIR to the SWIR [12]. Remarkably, Ti_3_C_2_T*_x_* enables plasmon wavelengths compressed to 1/100 of the free‐space wavelength in the SWIR [14], providing deep‐subwavelength confinement over an ultrabroad spectral range. While such confinement at high frequencies holds great promise for SEIRA, the potential of MXenes for broadband infrared molecular fingerprinting remains unexplored.

In this study, we introduce a new class of ultrabroadband SEIRA based on acoustic MXene plasmon (AMP) modes in vertically coupled Ti_3_C_2_T*_x_*–analyte–metal resonators, designed to overcome the spectral and sensitivity limitations of conventional 2D plasmonic systems. Leveraging the high carrier density of Ti_3_C_2_T*_x_*, the AMP structure supports resonance modes that not only extend into the SWIR, reaching frequencies up to 6866 cm^−1^, but also span an unprecedented spectral bandwidth of approximately 5000 cm^−1^. This combination of ultrabroadband operation and extreme plasmon confinement enables the detection of weak high‐frequency vibrational features—such as CH_3_ combination bands near 4700 cm^−1^—in molecular films as thin as 8 nm, with sensitivity enhancements far beyond those of conventional noble‐metal‐ or 2D‐material‐based SEIRA. These results establish acoustic plasmons in MXene as a powerful platform for next‐generation molecular sensing, combining ultrabroad spectral coverage with high‐resolution chemical sensitivity across the mid‐ and short‐wave infrared ranges.

## 2 Results

**FIGURE 1:**
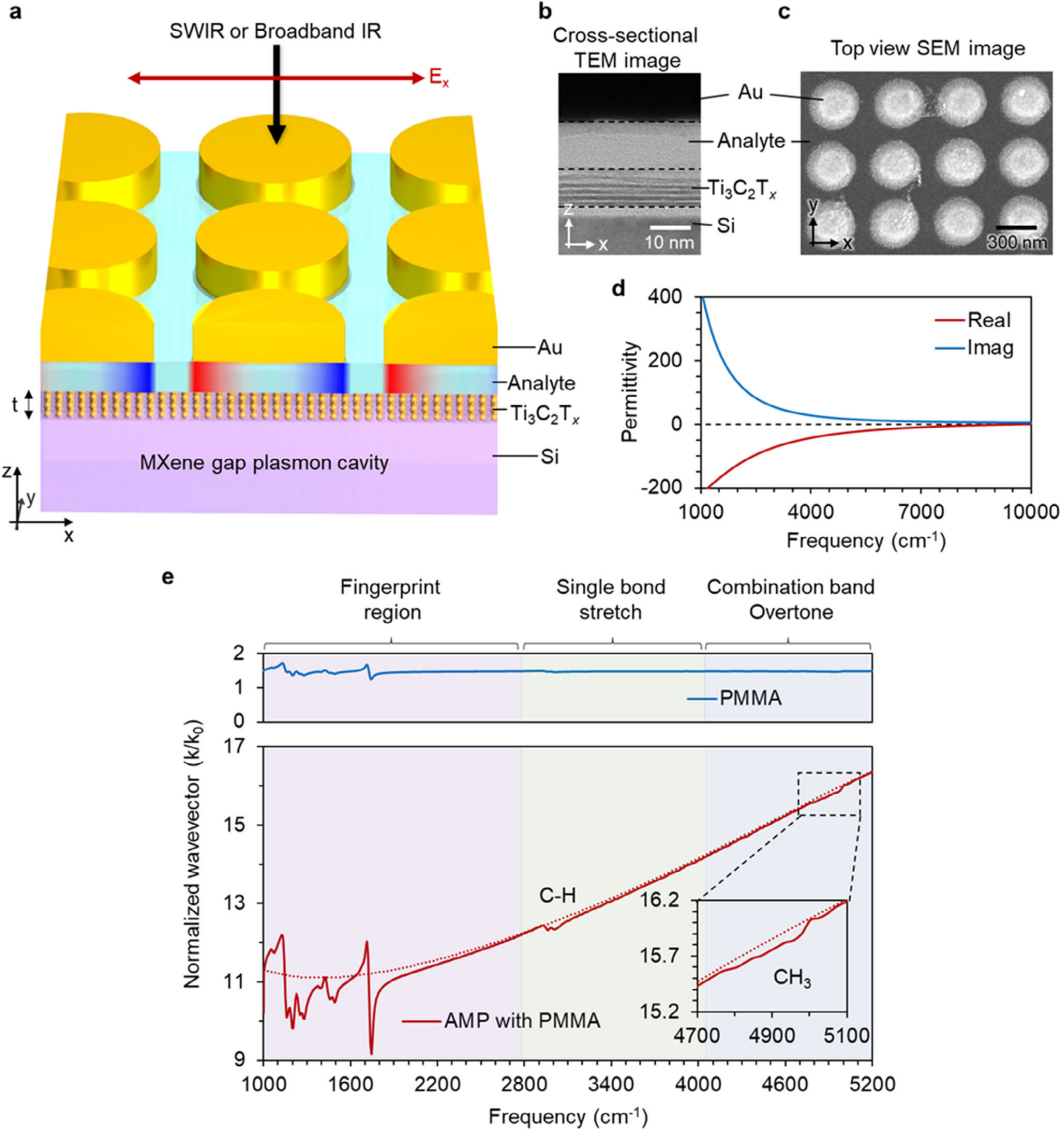
Acoustic MXene plasmon (AMP) resonator for broadband SEIRA. (a) Schematic illustration of the vertically coupled AMP resonator comprising Au nanodisks, an ultrathin analyte layer, a Ti_3_C_2_T*_x_* MXene film (thickness *t*), and a silicon substrate. The configuration supports acoustic plasmon modes confined within the vertical dielectric gap. (b) Cross‐sectional TEM image of the multilayer structure (Au–analyte–Ti_3_C_2_T*_x_*–Si) confirming sub‐10 nm uniformity of the MXene layer. (c) SEM image of the fabricated Au nanodisk array patterned atop the AMP cavity. (d) Measured complex permittivity (ε′, ε′′) of a 10 nm‐thick Ti_3_C_2_T*_x_* film via infrared ellipsometry. (e) Simulated dispersion relation of the AMP wavevector *k* normalized to the free‐space wavevector *k_0_* as a function of frequency, for an 8 nm PMMA analyte (bottom, red), compared to the constant‐index case (grey), and overlaid with the PMMA refractive index profile (top, blue), revealing strong plasmon‐analyte coupling and dispersion shifts at multiple vibrational bands, including ─C─H and ─CH_3_ combination modes in the SWIR.

We designed a vertically coupled multilayer plasmonic resonator array based on AMPs, illustrated in Figure 1a, to enable a SEIRA with high sensitivity, broad spectral bandwidth, and access to high‐frequency vibrational signatures. Our AMP device consists of a layered structure comprising a MXene (Ti_3_C_2_T*_x_*) film and a gold (Au) nanodisk array, separated by an ultrathin analyte layer on a Si substrate. In this configuration, the plasmons in the MXene layer strongly couple with their image charges induced in the Au nanostructures, giving rise to vertically confined acoustic plasmon modes that localize electromagnetic energy tightly within the analyte gap, enabling strong field confinement particularly in the SWIR region [14]. Unlike conventional localized surface plasmon resonances of isolated metallic nanoparticles, this mode originates from the hybridization between the MXene plasmon and its image charges in the metal resonator across the nanoscale gap. The AMP mode supported by this MXene – dielectric analyte – Au metal structure exhibits an ultrashort effective plasmon wavelength, confined by more than two orders of magnitude relative to the free‐space wavelength, resulting in intense near‐field enhancement and efficient coupling with molecular vibrational modes. Such strong field confinement amplifies even intrinsically weak vibrational signatures, making the structure ideally suited for high‐sensitivity SEIRA applications [1, 4].

To experimentally implement the AMP resonator array, we employed approximately 10 nm‐thick films of Ti_3_C_2_T*_x_* MXene, synthesized via a minimally intensive layer delamination (MILD) method by selectively etching the aluminum layer from Ti_3_AlC_2_ MAX phase in an aqueous acidic solution (see Methods). Cross‐sectional transmission electron microscopy (TEM) images (Figure 1b) reveal the multilayered structure of Ti_3_C_2_T*_x_*, with periodically spaced Ti_3_C_2_T*_x_* layers and subnanometer interlayer spacings. The chemical states of the MXene film were further confirmed by X‐ray photoelectron spectroscopy (XPS) (see Note S1). On top of the MXene layer, we fabricated the AMP resonator structure using standard nanofabrication techniques, including thin‐film deposition, electron‐beam lithography, and either lift‐off or ion milling, as illustrated in Figure 1c (see Methods). To evaluate the optical properties of the 10 nm‐thick Ti_3_C_2_T*_x_* layer, we characterized its complex permittivity across the infrared spectrum using spectroscopic ellipsometry, as shown in Figure 1d. The extracted permittivity reveals a high plasma frequency (ω_*p*_) of approximately 9700 cm^−1^ (1.20 eV), corresponding to the condition where the real part of the permittivity (ε′) crosses zero. This yields a surface plasmon frequency, given by $\omega_{sp}=\omega_{p}\;/\sqrt{2}$, of approximately 6860 cm^−1^ (0.85 eV), which is substantially higher than those of conventional 2D plasmonic materials such as graphene or black phosphorus, enabling access to high‐frequency vibrational modes in the SWIR regime. The elevated plasma frequency arises from the intrinsically high carrier density (*n*_2*D*_) of Ti_3_C_2_T*_x_* with a typical value of 7.5 × 10^14^ cm^−2^ as described by [22]:

(1) $$\omega_{p}\propto \sqrt{\frac{n_{2D}e^{2}}{\varepsilon_{0}m^{*}}}$$

where *e* is the elementary charge, ε_0_ the vacuum permittivity, and *m* ^∗^ the effective electron mass. The combination of high carrier density and metallic conductivity in Ti_3_C_2_T*_x_* MXene enables a plasmon mode in the SWIR regime, allowing access to high‐frequency molecular vibrational modes that are typically inaccessible with other 2D plasmonic materials. The spectral range investigated in this work lies well below the interband transition energies of Ti_3_C_2_T*_x_*, ensuring that the optical response is dominated by intraband plasmonic excitations.

Based on the measured optical properties, we analytically solved the plasmon dispersion relation for the full multilayer system, such as Si/Ti_3_C_2_T*_x_*/PMMA/Au (see Note S2) [23]. For the analysis, we modeled the structure as a multilayer system consisting of a semi‐infinite Au layer, an 8 nm‐thick PMMA, and a 10 nm‐thick Ti_3_C_2_T*_x_* film on a Si substrate. The refractive index of PMMA in the infrared range was taken from established literature values [24] and depicted in Figure 1e. As plotted in Figure 1e, the AMP mode exhibits distinct sensitivity to refractive index perturbations over a broad infrared spectral range, including the high‐frequency vibrational region (> 4000 cm^−1^) that is difficult to measure using conventional SEIRA due to limited field confinement and weak spectral coupling to overtone and combination bands. Notably, although the intrinsic refractive index variation of amorphous PMMA film is too small to be detected in the SWIR range owing to the inherently weak oscillator strength of these high‐frequency vibrational modes, the AMP resonance exhibits distinct dispersion shifts, indicating strong light–matter interaction with molecular fingerprints such as —CH_3_ combination bands. This is attributed to the extremely enhanced field confinement near the surface plasmon frequency of Ti_3_C_2_T*_x_*, which leads to a substantial increase in the effective refractive index (i.e., normalized wavevector, *k*_AMP_/*k*_0_). These results highlight the exceptional capability of AMP resonators for amplifying weak molecular vibrational signals beyond the spectral reach of existing SEIRA.

**FIGURE 2:**
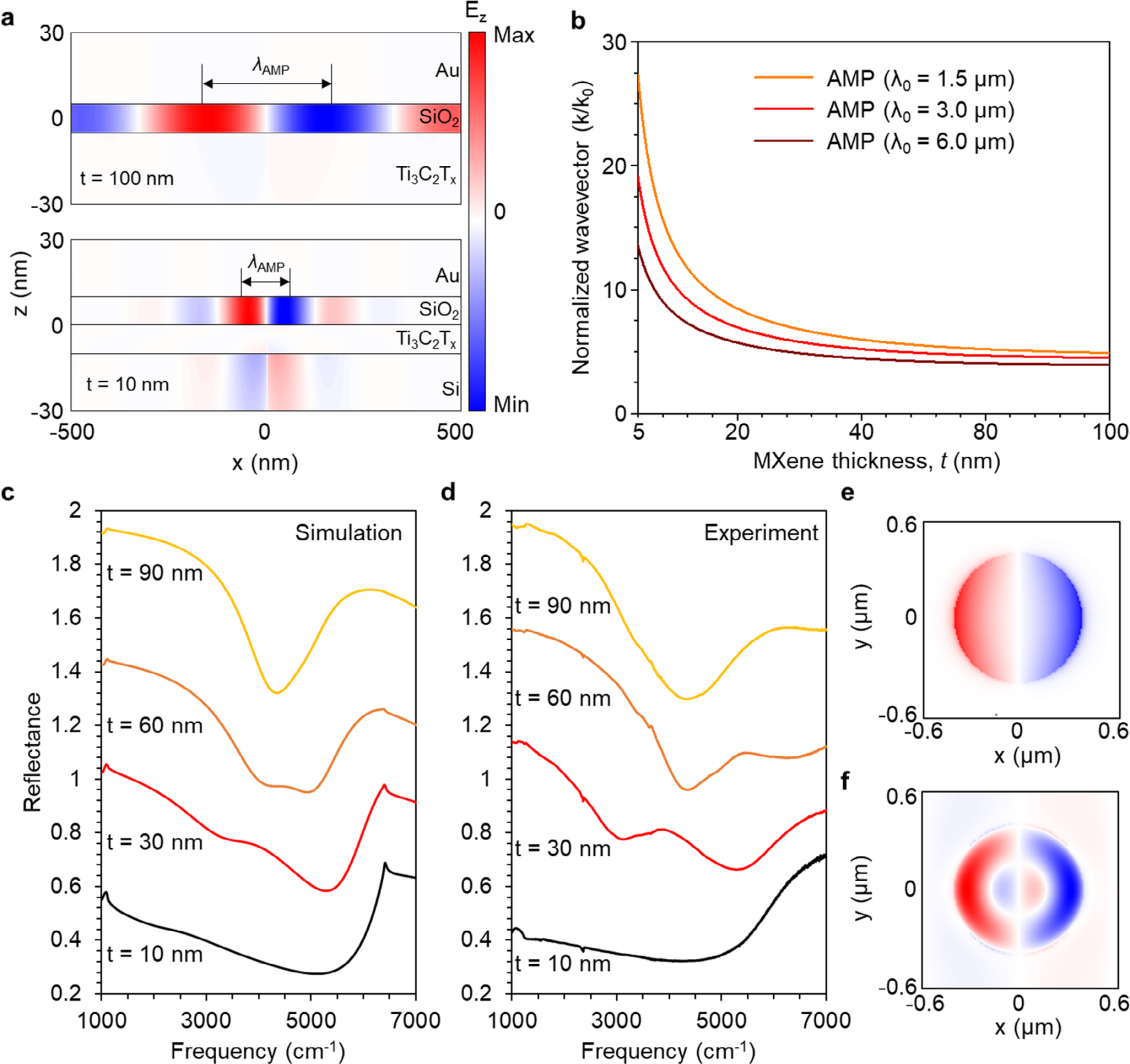
MXene thickness–dependent plasmon confinement and broadband response in AMP resonators. (a) Simulated electric field distribution (*E*_z_) of the AMP mode at a free‐space wavelength of 3 µm for Ti_3_C_2_T*_x_* thicknesses of 100 nm (top) and 10 nm (bottom), showing vertically confined, antisymmetric plasmonic fields propagating bidirectionally along the dielectric gap. (b) Extracted effective refractive index (i.e., normalized wavevector, *k*/*k*_0_) as a function of MXene thickness (*t)* at various wavelengths, indicating enhanced plasmon confinement with thinner films. (c, d) Simulated (c) and measured (d) reflectance spectra of AMP resonators with varying MXene thicknesses (*t*) (10, 30, 60, and 90 nm), revealing spectral redshifts and resonance broadening with increasing confinement. (e, f) Electric field distributions of the AMP resonator showing the (1,1) fundamental mode (e) and the (2,1) higher‐order resonance (f) at 10 nm Ti_3_C_2_T*_x_* thickness, corresponding to the eigenmodes of circular nanodisk arrays.

To understand the confinement mechanism in AMP resonators and identify key parameters for optimizing SEIRA performance, we systematically investigated the influence of the Ti_3_C_2_T*_x_* MXene thickness (*t*) on plasmonic field confinement and modal properties. Figure 2a presents the simulated electric field profiles of the AMP mode at a free‐space wavelength of 3 µm (3333 cm^−1^), revealing pronounced wavelength compression with field confinement. The AMP mode is characterized by an antisymmetric electric field distribution, with strong energy localization in the dielectric gap due to the charge‐coupling effect between the Au nanostructure and the MXene film [4, 14]. For a 100 nm‐thick MXene layer, the effective refractive index is 4.36 + 0.8i, whereas reducing the thickness to 10 nm significantly enhances the field confinement, yielding a much larger effective refractive index of 11.82 + 5.64i. This trend is further quantified in Figure 2b, which shows the effective refractive index as a function of *t* for various wavelengths. At *λ*_0_  =  1.5 µm, the effective refractive index exceeds 29 for *t*  =  5 nm, while it drops below 10 for *t*  >  40 nm. For longer wavelengths (e.g., *λ_0_ * =  3.0 µm and 6.0 µm), the effective refractive index decreases across all thicknesses, indicating weaker plasmon confinement at lower frequencies. These results confirm that a thinner MXene film supports stronger field confinement with a high effective refractive index, which is essential for achieving a broad spectral range and enhanced light–matter interaction in AMP‐based SEIRA. To further verify that this thickness‐dependent confinement mechanism remains valid in the presence of an analyte layer, we performed additional simulations using PMMA as the dielectric layer, which show consistent dispersion behavior (see Note S3).

Building on the theoretical and simulation‐based analysis of AMP field confinement, we next experimentally investigated the resonance spectrum of the AMP resonator—namely, its ability to support ultrabroadband resonances and give access to vibrational modes in the high frequency range, including the SWIR regime. Consequently, we fabricated a series of AMP resonators with fixed lateral geometries: 40 nm‐thick Au disks with a diameter *D* = 300 nm and periodicity *L* = 450 nm, separated by a 10 nm‐thick SiO_2_ spacer, while systematically varying only the MXene thickness (*t* = 10 nm, 30 nm, 60 nm, and 90 nm). Reflectance spectra were acquired using a Fourier‐transform infrared (FTIR) spectrometer (Nicolet iN10Mx, Thermo Scientific) equipped with a 15× microscope objective, covering the broad spectral range from 700 cm^−1^ to 7800 cm^−1^. A 200 nm‐thick Au‐coated silicon wafer was used as the background reference. Figure 2c,d presents the simulated and experimentally measured reflectance spectra, respectively, for different MXene thicknesses. Two dominant resonance features are observed: the fundamental (1,1) mode and a higher‐order (2,1) mode, as shown in Figure 2e,f, respectively. These plasmonic resonances correspond to angular and radial eigenstates of circular cavities, described by eigenfunctions $\phi_{n,m}\propto J_{n}\left(\;\;\;\rho \;\;\;\right)\exp\left(im\phi \right)$, where *J*_n_ is the Bessel function of the first kind, *ρ* is the radial coordinate, ϕ is the azimuthal angle, *n* denotes the radial mode number, and *m* is the azimuthal mode number indicating the number of angular nodes. For thick MXene films (*t* = 90 nm), the (1,1) mode dominates due to the longer plasmon wavelength and weak coupling efficiency of higher‐order modes. As the MXene thickness is reduced, the (2,1) mode is more clearly observed, and resonant frequencies redshift due to enhanced field confinement. Remarkably, at *t* = 10 nm, the two resonances spectrally overlap, forming a broad and deep reflectance dip. This spectral broadening arises from increased modal damping (i.e., a larger imaginary component of the plasmon wavevector, *Im*(*k*_AMP_)), enabling broadband operation over the entire MIR range within a single resonator. Such multimodal hybridization is particularly advantageous for SEIRA, as it increases spectral overlap with a diverse set of molecular vibrational fingerprints.

**FIGURE 3:**
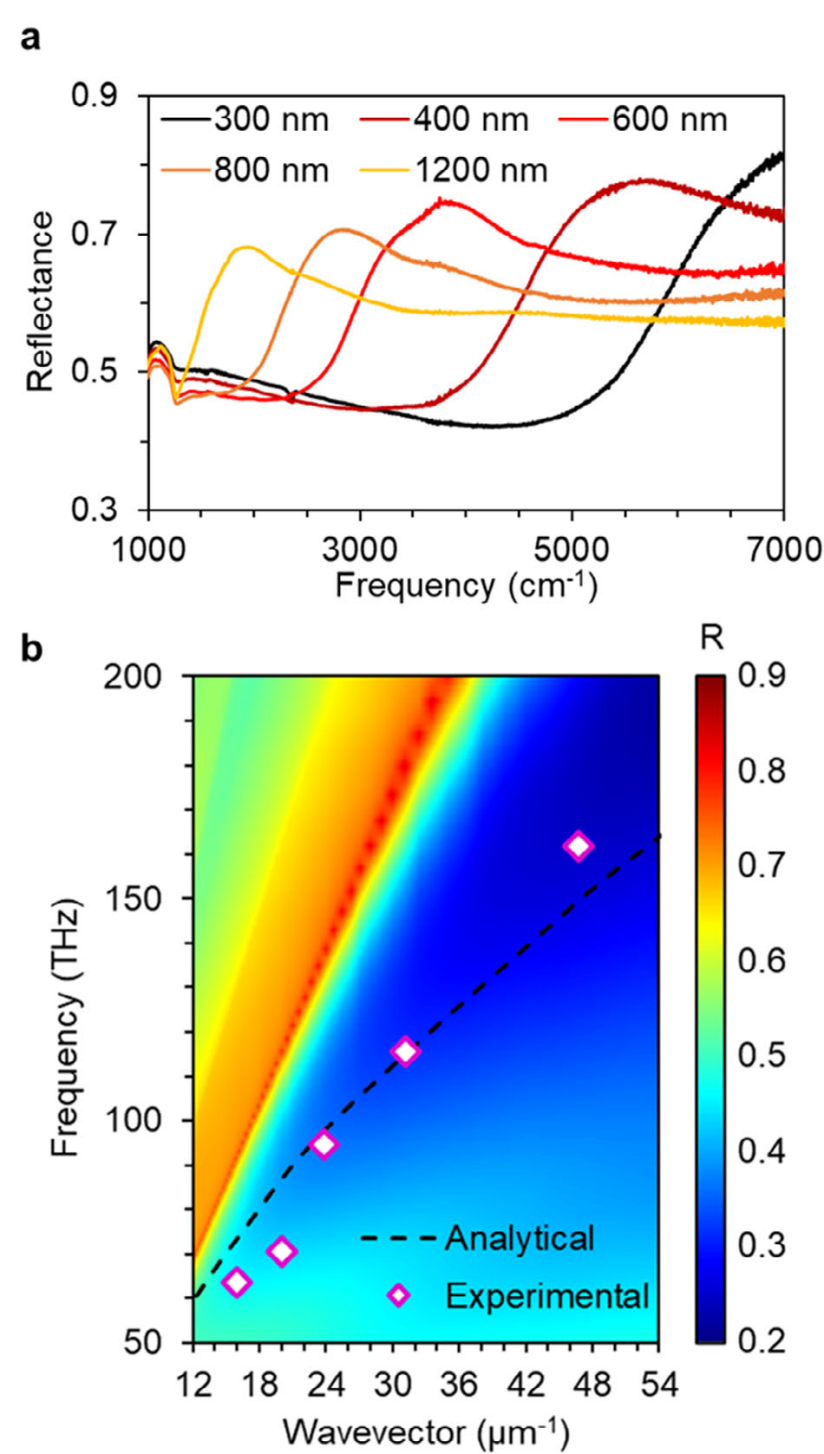
Experimental verification of AMP dispersion via diameter‐dependent spectral response. (a) Measured reflectance spectra of AMP resonator arrays with varying Au nanodisk diameters (300–1200 nm), showing systematic redshifts of the resonance dip as disk size increases. (b) Extracted dispersion relation of the AMP mode. The experimental resonance frequencies (magenta diamonds) are plotted as a function of the effective in‐plane wavevector *k*_p_, estimated from resonator diameter *D* using the relation *k*_p_ = [2*x*_2_(*J*_1_(*x*)) −arg(*r*)]/*D*, where arg(*r*) ∼ 0.9π corresponds to the (2,1) resonance condition. The color map shows numerically calculated reflectance spectra, and the dashed black curve denotes the analytically predicted AMP dispersion, demonstrating strong agreement with experimental observations.

To confirm that these broadband reflectance dips are related to the AMP dispersion, we further investigated the spectral tunability of the resonance by varying the disk diameter *D* from 300 nm to 1200 nm, while fixing the MXene and spacer thicknesses at 10 nm. The resonance condition is governed by the lateral size of the Au disk, which determines the resonance frequency of the confined AMP mode. The disk periodicity was set to *L*_x_ = *L*_y_ = 1.5*D* to ensure consistent inter‐resonator spacing (see Note S4). The measured reflectance spectra, shown in Figure 3a, exhibit a clear redshift of the resonance frequency as *D* increases, consistent with the dispersive behavior of the AMP mode: larger resonators support lower in‐plane wavevectors with a low spatial frequency, thus shifting the resonance to lower frequencies. All measured spectra also exhibit a small reflection dip near 1200 cm^−1^, attributed to coupling between the AMP mode and the transverse optical phonon mode in the SiO_2_ layer. The dominant absorption peak at each diameter arises from the (1,1) mode, which overlaps with the (2,1) mode due to spectral broadening, as previously discussed in Figure 2. To evaluate these experimental observations with theoretical expectations, we obtained the resonance frequencies and plotted them against the corresponding in‐plane wavevectors [25], comparing the results to both analytical dispersion curves and numerical simulations (see Note S2). To further clarify the plasmonic origin of the resonances and the role of the Au resonator in the AMP structure, we performed additional simulations comparing bare Au disks and the AMP resonator (see Note S5). These results demonstrate that the Au‐resonator‐enabled AMP mode leads to strong field confinement, resulting in enhanced SEIRA sensitivity. As shown in Figure 3b, the experimental data (pink diamonds) are in close agreement with the theoretically calculated AMP dispersion relation (black dotted line). This good agreement between experiment, theory, and simulation confirms that the observed resonances are associated with cavity modes of the AMP. These results demonstrate that the AMP resonator supports ultrabroadband, highly confined plasmonic modes with precise spectral tunability and access to high‐frequency regions, including the SWIR, validating its robustness as a next‐generation SEIRA.

**FIGURE 4:**
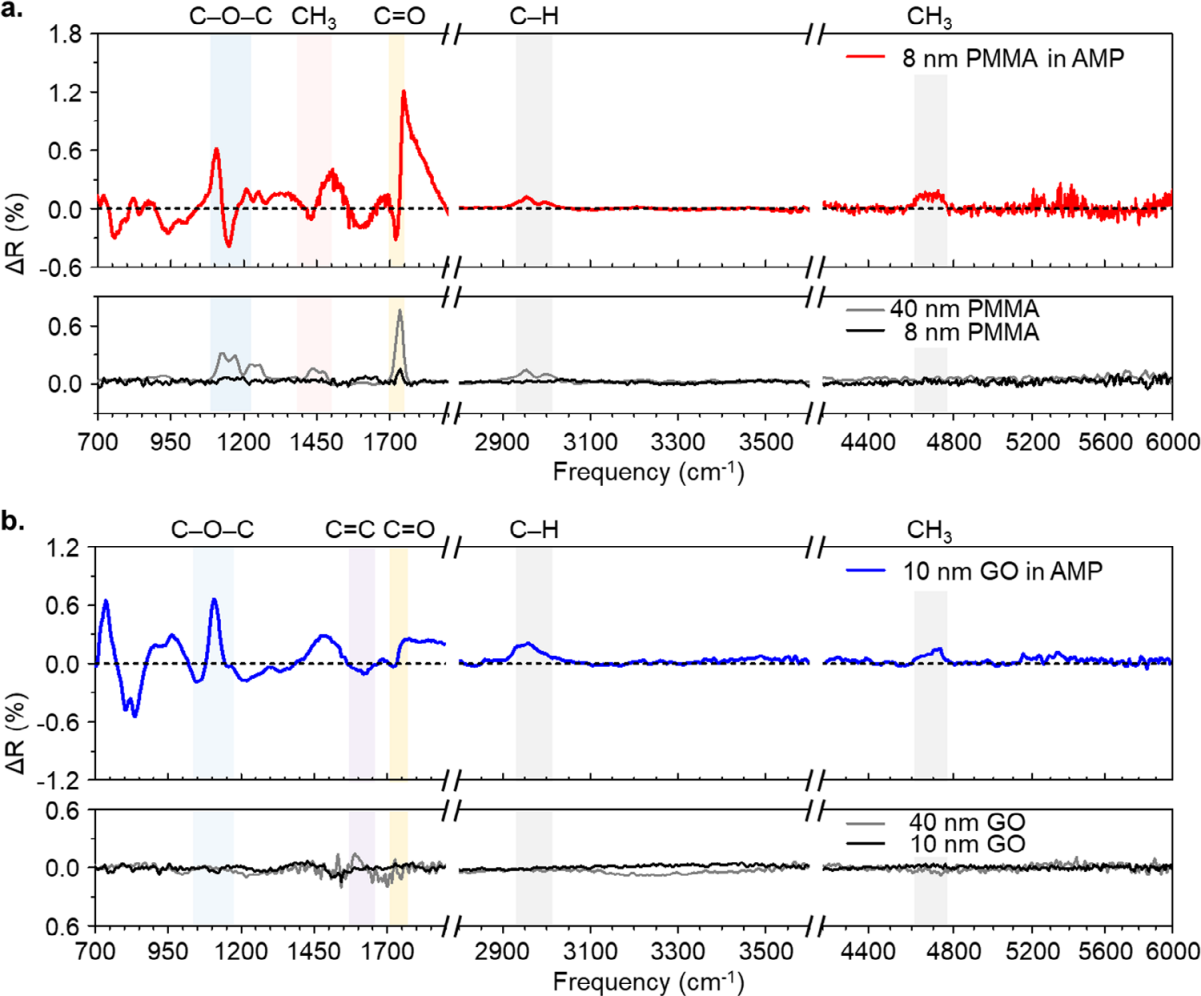
Ultrabroadband SEIRA spectra of ultrathin analyte films using AMP resonators. (a) SEIRA spectrum of an 8 nm‐thick PMMA film measured using an AMP array with 1100 nm‐diameter Au nanodisks (top, red). Multiple vibrational fingerprints–including C─O─C stretching (∼1150 cm^−1^), CH_3_ bending (∼1435 cm^−1^), C═O stretching (∼1730 cm^−1^), C─H stretching (∼2950 cm^−1^), and CH_3_ combination bands (∼4700 cm^−1^), are clearly resolved across the MIR–SWIR range. Reference spectra from 8 nm‐ and 40 nm‐thick PMMA films (bottom, black and gray) without enhancement show negligible signal. (b) SEIRA spectrum of a 10 nm‐thick GO film acquired using an AMP array with 1000 nm‐diameter Au nanodisks (top, blue), revealing C─C─O stretching (∼1150 cm^−1^), C═C stretching (∼1620 cm^−1^), C═O stretching (∼1730 cm^−1^), C─H stretching (∼2950 cm^−1^), and CH_3_ combination bands (∼4700 cm^−1^). Reference spectra from 10 nm‐ and 40 nm‐thick GO films (bottom, black and gray) exhibit no discernible vibrational features.

To experimentally validate the capability of our AMP resonator for ultrasensitive broadband molecular fingerprinting, we applied it to detect vibrational signatures in two ultrathin analyte films: an 8 nm‐thick PMMA layer and a 10 nm‐thick graphene oxide (GO) film. Both contain vibrational modes with intrinsically weak oscillator strengths, particularly in SWIR regions. Figure 4 presents the SEIRA spectra obtained using AMP resonators optimized for each analyte (see Methods and Note S6). To extract the molecular vibrational signals from the background response of the AMP resonances, we fitted the plasmonic response without perturbation by the vibrational signal with a Lorentzian curve. We obtained the deviation by subtracting the fitted results from the measurement results (see Note S7). In Figure 4a, the SEIRA spectrum (top, red trace) was acquired from an AMP resonator composed of a 10 nm‐thick Ti_3_C_2_T*_x_* MXene film and 40 nm‐thick Au nanodisks with a diameter of 1100 nm, designed to support a broad resonant bandwidth. Despite the analyte being only 8 nm‐thick PMMA, our device could detect multiple vibrational signatures spanning from 700 cm^−1^ to 6000 cm^−1^ in a single measurement, which is enabled by the ultrabroadband nature of the AMP‐enhanced SEIRA. Specifically, clear absorption peaks corresponding to the C─C and C─O stretching modes (∼1150 cm^−1^), CH_3_ bending (∼1435 cm^−1^), C═O stretching (∼1730 cm^−1^), and C─H stretching (∼2950 cm^−1^) are observed. Most notably, a weak CH_3_ combination band near 4700 cm^−1^ located at the SWIR region is resolved with high signal fidelity, an area typically inaccessible to conventional SEIRA. In contrast, the reference spectra measured from bare PMMA films without AMP enhancement (black trace for 8 nm, gray trace for 40 nm) reveal that even with five times thicker, only a limited set of mid‐infrared vibrational features emerge, and all high‐frequency modes above 3000 cm^−1^ remain undetectable. This direct comparison clearly shows the critical role of AMP‐induced strong field confinement in amplifying vibrational signals that are otherwise below the detection limit in conventional systems, especially in the SWIR. Simulations further confirm that the molecular vibrational signal cannot be resolved in the bare Au resonator due to weak electromagnetic confinement, whereas the AMP resonator clearly detects the SEIRA signal (see Note S8).

Similarly, Figure 4b presents the SEIRA spectrum obtained from a 10 nm‐thick GO film using an AMP resonator array consisting of 1000 nm‐diameter Au nanodisks on top of a 10 nm Ti_3_C_2_T*_x_* MXene layer. The chemical structure of the GO film was further confirmed by XPS (see Note S9). The measured spectrum reveals multiple distinct vibrational features that are absent in the reference signal from a bare 10 nm‐thick film (black trace). In particular, a prominent C─H stretching vibration is observed near 2950 cm^−1^, accompanied by a broad absorption band centered around 3500 cm^−1^ attributed to O─H stretching modes, which are characteristic of surface‐bound hydroxyl groups and interlayer water molecules in GO. Notably, a weak yet clearly resolvable vibrational feature emerges near 4700 cm^−1^, a spectral region typically dominated by vibrational combination and overtone modes with extremely weak vibrational signals. We attribute this high‐frequency signal to a CH_3_ combination band. This feature has not been previously observed in ultrathin GO layers due to its negligible absorption and minimal refractive index modulation (Δ*n* ≪ 0.01) [26]. Even more remarkably, the spectrum exhibits a distinct signal near 5200 cm^−^^1^, extending into an exceptionally high‐frequency regime that has remained unexplored in conventional SEIRA, underscoring both the ultrabroadband detection capability of the AMP resonator and its unprecedented access to high‐frequency vibrational features. It is noteworthy that Ti_3_C_2_T*_x_* MXene exhibits good chemical and thermal stability [27, 28]. Subsequently, our AMP devices show no noticeable degradation in the plasmonic resonance after several months of storage under ambient conditions (see Note S7 and Figure S9).

## 3 Discussion

**FIGURE 5:**
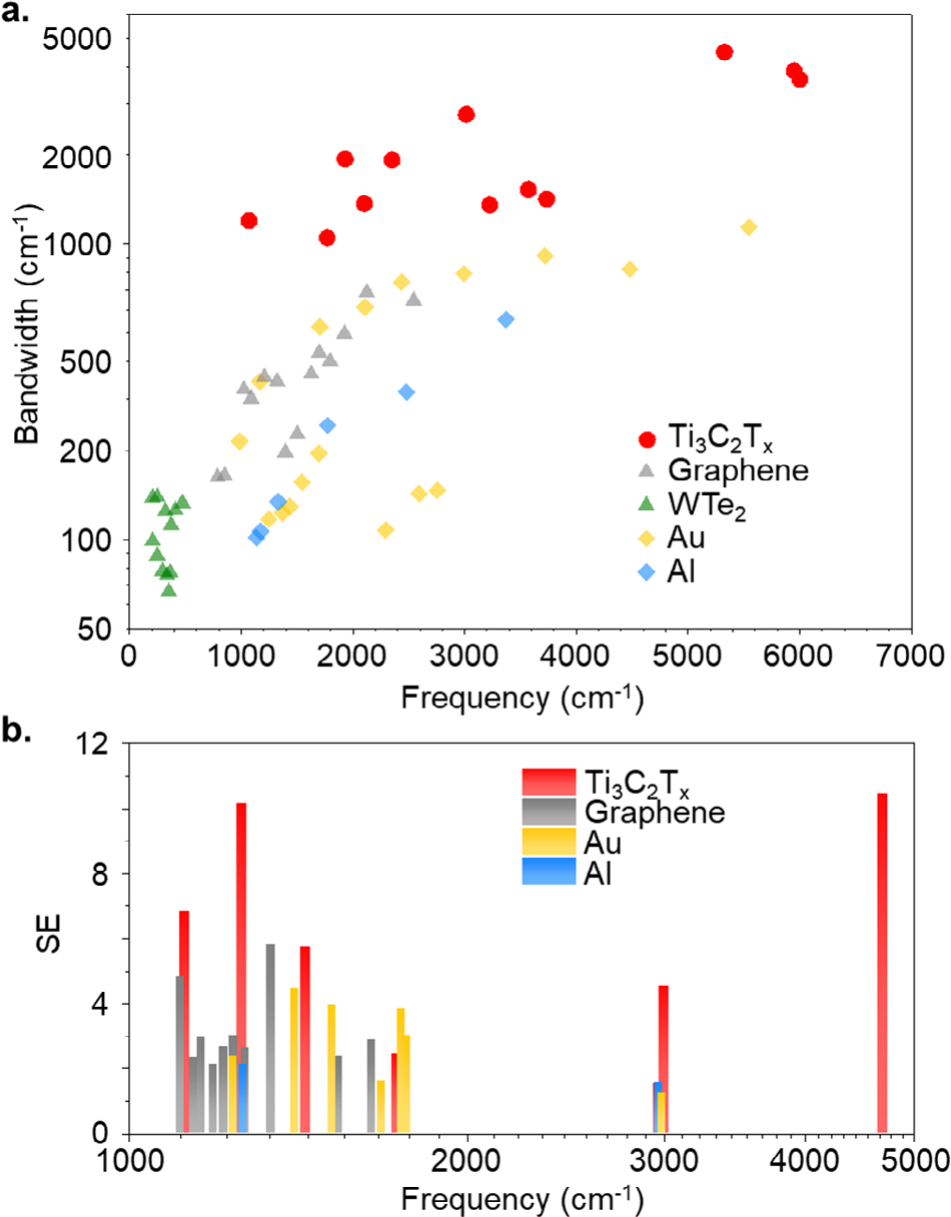
Comparative performance of SEIRA based on various plasmonic materials. (a) Bandwidths of resonators based on noble metals (gray), graphene (green), WTe_2_ (blue), and Ti_3_C_2_T*_x_* MXene (red; this work). The AMP resonator exhibits an ultrabroadband response (>4000 cm^−1^), surpassing previously reported systems typically limited to <1100 cm^−1^. (b) SE factors for representative vibrational modes across different analytes. The AMP resonator outperforms noble metal and graphene resonators, particularly in high‐frequency regions (>3000 cm^−1^), demonstrating superior light–matter interaction and broadband detection capability.

In Figure 5a, we summarize the bandwidth of reported plasmon resonators based on 2D materials and noble metals, which is associated with SEIRA performance. Overall, the plasmon resonators with noble metals [8, 29, 30] or 2D materials including graphene [1, 2, 3, 4], and WTe_2_ [31], show narrow spectral bandwidths, typically below ∼1100 cm^−1^ in MIR range. In particular, WTe_2_, despite its intrinsic in‐plane anisotropy, operates primarily in the far‐infrared range with narrowband resonances (∼200 cm^−1^) due to its low surface plasmon frequency, making it unsuitable for MIR applications where most molecular fingerprints exist. For the case of graphene resonators, while they support strong plasmon confinement in the MIR range, they cannot support plasmon modes in a frequency range higher than 3300 cm^−1^ due to their low carrier density. Meanwhile, metal‐based SEIRA devices, although benefiting from high carrier concentrations of noble metal, suffer from weak field confinement, especially in low‐frequency regions.

By contrast, our Ti_3_C_2_T*_x_* MXene‐based AMP resonators support an ultrabroad plasmonic response spanning from the far‐infrared to the SWIR, enabled by a high surface plasmon frequency. This allows access to vibrational modes across a wide spectral window from 1100 cm^−1^ to beyond 5000 cm^−1^, encompassing both major vibrational signatures in the mid‐IR range and higher‐order overtone and combination bands in the SWIR. This broad operating frequency arises from modal damping associated with strong field confinement with short plasmon wavelength.

To quantify the sensitivity of SEIRA performance, we evaluated the sensitivity enhancement (SE) factor, defined as:

(2) $$SE=\frac{\Delta I_{\mathrm{deivce}}}{\Delta I_{\mathrm{film}}}$$

where Δ*I*_device_ denotes a change of reflectance by vibrational signature in the AMP device, and Δ*I*_film_ is the absorption by film due to vibrational signature given by Δ*I*_film_  =  1 − exp(−α*∙ g*); α is the absorption coefficient at the vibrational frequency and *g* is the thickness of the analyte layer at a given wavelength.

As summarized in Figure 5b, our AMP‐based SEIRA exhibits SE values that exceed those of both graphene‐ and metal‐based SEIRA across a range of molecular vibrations. For example, in PMMA, we obtained SE values of 2.7 at 1150 cm^−1^ (C─C/C─O stretching), 2.6 at 1435 cm^−1^ (CH_3_ bending), 2.6 at 1730 cm^−1^ (C═O stretching), and 1.8 at 2962 cm^−1^ (C─H stretching). Remarkably, a SE of 10 was achieved at 4700 cm^−1^ for the CH_3_ combination band, a spectral region typically inaccessible due to extremely weak vibrational cross‐sections (Δ*k* = 0.0016). Similarly, for GO, the AMP resonator yielded SE values of 5.3 at 1120 cm^−1^ and 5.8 at 2962 cm^−1^ and further enabled the detection of high‐frequency vibrational signals at 4700 cm^−1^ that were undetectable in bare films.

## 4 Conclusions

We have demonstrated surface‐enhanced infrared absorption (SEIRA) spectroscopy using the acoustic MXene plasmons (AMP) of Ti_3_C_2_T*_x_*. By exploiting the high carrier density and ultrathin geometry of MXene films, the proposed AMP resonator exhibits two orders of plasmon wavelength reduction with an unprecedented spectral bandwidth exceeding 5000 cm^−1^, spanning from the LWIR to the SWIR. This broadband response enables simultaneous detection of vibrational signatures spanning from the low‐frequency to high‐frequency regime, which are typically inaccessible with conventional graphene‐based or metal‐based SEIRA. We demonstrated the high performance of this system through the detection of weak vibrational fingerprints in nanometer‐scale analyte films, including PMMA and graphene oxide, achieving a sensitivity enhancement higher than 10 for the weak CH_3_ combination modes in the SWIR regime. These results validate the AMP resonator's ability to break the traditional trade‐off relationship between sensitivity and bandwidth, establishing a new paradigm for ultrathin‐film molecular spectroscopy.

Our AMP platform for characterization of molecular behavior with broad bandwidth and strong plasmon confinement opens new directions for advanced chemical sensing, including trace analyte detection, surface‐enhanced Raman spectroscopy, and spectroscopic imaging at the nanoscale. Furthermore, the integration of MXene‐based AMP resonators into on‐chip platforms holds promise for a broad range of MIR–SWIR photonic applications, including compact infrared detectors, modulators, and real‐time biochemical sensors. These findings could show the potential of acoustic MXene plasmons as a foundational building block for the next generation of broadband, ultrasensitive deep‐infrared nanophotonic systems.

## 5 Experimental Section/Methods

### 5.1 Synthesis and Deposition of Ti_3_C_2_T*_x_* MXene Films

Ti_3_C_2_T*_x_* MXene was synthesized via the minimally intensive layer delamination (MILD) method [32] by selectively etching the aluminum layers from Ti_3_AlC_2_ MAX phase powder (particle size ∼40 µm, Carbon‐Ukraine). Initially, 3.2 g of lithium fluoride (LiF, Alfa Aesar) was dissolved in 40 mL of 9 m hydrochloric acid (HCl, Sigma‐Aldrich) in a polypropylene bottle under continuous stirring. Subsequently, 2.0 g of Ti_3_AlC_2_ powder was slowly added to the solution, and the reaction proceeded at 35°C for 25 h under constant agitation. After etching, the mixture was repeatedly washed with deionized water and centrifuged at 3500 rpm for 5 min per cycle until the supernatant reached a pH of approximately 6, yielding a stable dispersion of delaminated Ti_3_C_2_T*_x_* nanosheets. To improve dispersion homogeneity, the resulting colloidal solution was argon‐purged and bath‐sonicated (LK‐U065D, 150 W) for 30 minutes under Ar flow. The solution was then centrifuged at 11 000 rpm for 30 min, the supernatant discarded, and the remaining paste redispersed in deionized water. The final concentration of the resulting MXene solution was approximately 45 mg mL^−^^1^. For film deposition, 10 nm‐thick Ti_3_C_2_T*_x_* layers were fabricated by spin‐coating the MXene solution onto silicon substrates. Prior to coating, the Si wafers were treated with UV‐ozone and functionalized by immersion in a 0.01 wt% solution of 3‐aminopropyltrimethoxysilane (APTMS). A volume of 0.6 mL of MXene solution was dispensed onto the substrate and spin‐coated at 7000 rpm for 10 s, followed by 5000 rpm for 60 s. The coated samples were subsequently dried on a hot plate at 90°C for 30 s to complete the film formation.

### 5.2 Fabrication of AMP Resonators

To fabricate the AMP resonators, a 10 nm‐thick Ti_3_C_2_T*_x_* MXene layer was first spin‐coated onto a silicon wafer, followed by deposition of a 10 nm‐thick SiO_2_ layer via RF sputtering. For nanostructure patterning, a poly(methyl methacrylate) (PMMA C4) resist was spin‐coated at 4000 rpm onto the SiO_2_ layer and soft‐baked. Periodic circular patterns were then defined using electron beam lithography (EBL). After exposure, the resist was developed in a 3:1 mixture of methyl isobutyl ketone (MIBK) and isopropyl alcohol (IPA) for 80 s. Subsequently, a 2 nm‐thick aluminum (Al) adhesion layer and a 40 nm‐thick gold (Au) layer were deposited by thermal evaporation. Finally, the lift‐off process was carried out by immersing the sample in acetone under sonication to remove the remaining PMMA resist, resulting in a well‐defined Au nanodisk array atop the SiO_2_/MXene/Si stack.

### 5.3 Fabrication of an AMP Resonator for Sensing PMMA

To fabricate the AMP resonator for SEIRA sensing of PMMA, a 10 nm‐thick Ti_3_C_2_T*_x_* MXene film was first spin‐coated onto a silicon substrate, followed by the deposition of an ultrathin 8 nm PMMA analyte layer (see Note S6 for details on PMMA coating). A 40 nm‐thick Au film was then deposited on top by thermal evaporation to serve as the plasmonic layer. To avoid damaging the PMMA analyte during patterning, we employed a negative‐tone hydrogen silsesquioxane (HSQ) resist for EBL, which eliminates the need for a lift‐off process using acetone. After exposure, the HSQ patterns were developed in AZ 300 MIF developer. Subsequently, the Au film was patterned using argon ion milling to achieve a precise etch of the 40 nm‐thick Au layer. Finally, the residual HSQ resist was removed by immersion in buffered oxide etchant (BOE), completing the fabrication of the AMP resonator integrated with the nanometer‐thick PMMA analyte.

### 5.4 Fabrication of an AMP Resonator for Sensing GO

To fabricate the AMP resonator for SEIRA sensing of GO, a 10 nm‐thick Ti_3_C_2_T*_x_* MXene film was first deposited on a silicon substrate via spin‐coating. Subsequently, a 10 nm‐thick GO layer was uniformly spin‐coated on top of the MXene film. For plasmonic patterning, PMMA C4 resist was spin‐coated at 4000 rpm onto the GO layer, and periodic circular patterns were defined using EBL. The exposed samples were developed in a MIBK:IPA (3:1) solution for 80 s. Following pattern development, 2 nm of Al (as an adhesion layer) and 40 nm of Au were sequentially deposited by thermal evaporation. Finally, the patterned plasmonic structures were defined by a standard lift‐off process in acetone under sonication to remove the residual PMMA resist, completing the fabrication of the AMP resonator integrated with the ultrathin GO analyte.

### 5.5 Numerical Simulations

Numerical simulations were carried out using both the finite‐difference eigenmode (FDE) and finite‐difference time‐domain (FDTD) methods to analyze the plasmonic response of the AMP resonator. To simulate the propagation behavior of the acoustic plasmon mode shown in Figure 1e, the FDE solver was employed to calculate the eigenmode profiles of the hybrid AMP modes in the Au–PMMA–MXene configuration. The extracted eigenmodes were subsequently used as excitation sources at *x* = 0, launched bidirectionally with a π‐phase difference to emulate antisymmetric AMP excitation. Reflectance spectra and near‐field distributions were computed using the FDTD method. The dielectric functions for silicon, silicon dioxide, gold, and PMMA were obtained from experimentally reported values in the literature. To solve Maxwell's equations, periodic boundary conditions were applied in the *x*‐ and *y*‐directions. At the same time, perfectly matched layers (PMLs) were imposed in the z‐direction to suppress non‐physical reflections. A broadband plane‐wave source was used, covering a frequency range from 1000 cm^−1^ to 7000 cm^−1^, to capture the complete infrared response of the AMP resonator.

## Author Contributions

C.P., Y.G., C.M.K., and M.‐K.K. conceived the project and supervised the overall research. C.P. proposed the core physical concept, conducted theoretical analysis and simulations, and coordinated the experiments. J.K. performed device fabrication, optical measurements, and data analysis. N.‐R.P. conducted TEM imaging and contributed to structural and materials characterization. H.K. (Hyerim Kim) synthesized and characterized the Ti_3_C_2_T*_x_* MXene films. H. K. (Hyeju Kim) assisted in experimental procedures. All authors contributed to the writing and revision of the manuscript.

## Conflicts of Interest

The authors declare no conflicts of interest.

## Supporting information




**Supporting File**: advs75346‐sup‐0001‐SuppMat.pdf.

## Data Availability

The data that support the findings of this study are available from the corresponding author upon reasonable request.
