# Supplementary material for: Ultrabroadband and Highly Sensitive Short‐Wave Infrared Molecular Fingerprinting via Acoustic MXene Plasmons
Source: Adv Sci (Weinh). 2026 Apr 16;13(38):e75346. doi: 10.1002/advs.75346 (PMC13335539; doi:10.1002/advs.75346)
Supplement: Supplementary file 1 — Supporting File: advs75346‐sup‐0001‐SuppMat.pdf. [file ADVS-13-e75346-s001.pdf]

Supporting Information

Ultrabroadband and highly sensitive short-wave infrared molecular fingerprinting via acoustic MXene plasmons

Changhoon Park,† Jisung Kwon,† Nu-Ri Park, Hyerim Kim, Hyeju Kim, Yury Gogotsi, Chong Min Koo, and Myung-Ki Kim**

†These authors contributed equally: Changhoon Park, Jisung Kwon

Changhoon Park, Jisung Kwon, Nu-Ri Park, Hyeju Kim, Myung-Ki Kim
KU-KIST Graduate School of Converging Science and Technology, Korea University, Seoul 02841, Republic of Korea
E-mail: rokmk@korea.ac.kr

Changhoon Park, Yury Gogotsi
A. J. Drexel Nanomaterials Institute and Department of Materials Science and Engineering, Drexel University, Philadelphia, PA 19104, USA

Yury Gogotsi
Department of Materials Science and Engineering, Korea University, Seoul 02841, Republic of Korea

Hyerim Kim, Chong Min Koo
School of Advanced Materials Science & Engineering, Sungkyunkwan University, Suwon-si 16419, Republic of Korea
E-mail: chongminkoo@skku.edu

Chong Min Koo
School of Chemical Engineering, Sungkyunkwan University, Suwon-si 16419, Republic of Korea

Myung-Ki Kim
Department of Integrative Energy Engineering, College of Engineering, Korea University, Seoul 02841, Republic of Korea

Table of Contents

1. XPS characterization of $Ti_3C_2T_x$ MXene film 3

2. Dispersion analysis of acoustic MXene plasmons (AMPs) 4

3. Validation of thickness-dependent plasmon confinement using PMMA 6

4. Fabrication and SEM characterization of AMP resonators 7

5. Plasmonic response and field confinement in AMP resonators 8

6. Thickness control of ultrathin PMMA and GO films 9

7. Comprehensive SEIRA spectra and fitting analysis 10

8. Comparison of SEIRA responses between AMP and bare Au resonators 14

9. XPS characterization of graphene oxide (GO) film 15

Supplementary note 1. XPS characterization of $Ti_3C_2T_x$ MXene film

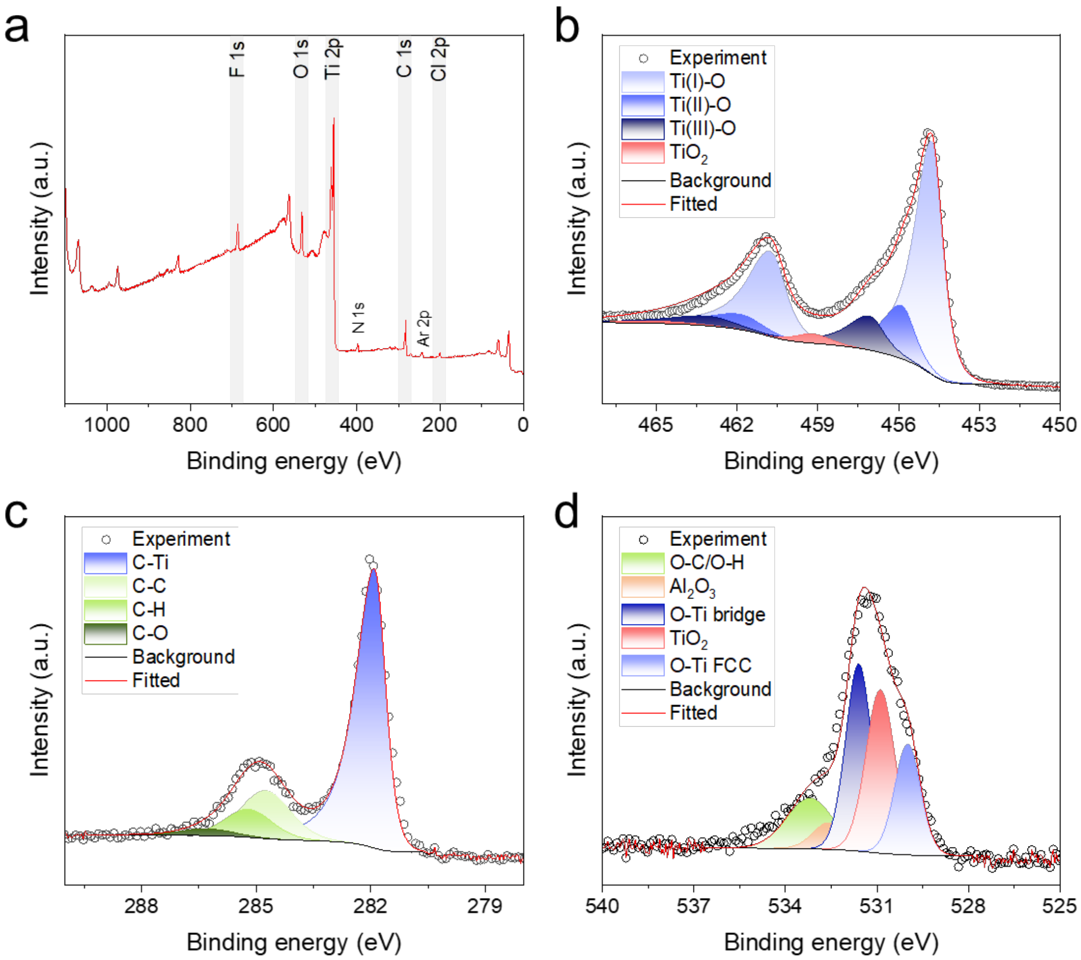

Figure S1. XPS characterization of $Ti_3C_2T_x$ MXene film. a Survey XPS spectrum confirming the elemental composition of the spin-coated $Ti_3C_2T_x$ film, and **b-d** high-resolution core level spectra of **b** Ti 2p, **c** C 1s, and **d** O 1s showing characteristic bonding states associated with Ti–C and surface termination groups in $Ti_3C_2T_x$ MXene. The fitted components indicate the presence of Ti–C bonding together with Ti–O related surface terminations, while no dominant signatures of fully oxidized TiO_2 are observed.

Supplementary note 2. Dispersion analysis of acoustic MXene plasmons (AMPs)

To obtain the dispersion relation of the acoustic MXene plasmon (AMP) mode, we analytically solved Maxwell's equation in four layers with the following method. For transverse-magnetic (TM) waves propagating in the *x*-direction, the *x* and *z* components of the electric field are given by

$$E_{x,n} = \left[a_n e^{\gamma_n z} + b_n e^{-\gamma_n z}\right] e^{i\beta x} \quad \text{(S1)}$$

$$E_{z,n} = \left[-\frac{i\beta}{\gamma_n} a_n e^{\gamma_n z} + \frac{i\beta}{\gamma_n} b_n e^{-\gamma_n z}\right] e^{i\beta x} \quad \text{(S2)}$$

where a_n and b_n are amplitude coefficients, and β and γ are in-plane wavevector and out-of-plane wavevector determined with a transcendental equation, $\beta^2 = \gamma_n^2 + \varepsilon_n k_0^2$. By applying field continuity to Equation (S1) and (S2) at each interface of layers, we derived eight coupled equations for a_n and b_n, which can be obtained with the following determinant being zero.

$$\det\begin{pmatrix} 0 & 1 & 0 & 0 & 0 & 0 & 0 & 0 \\ 1 & 1 & -1 & -1 & 0 & 0 & 0 & 0 \\ -\frac{\gamma_1}{\varepsilon_1} & \frac{\gamma_1}{\varepsilon_1} & \frac{\gamma_2}{\varepsilon_2} & -\frac{\gamma_2}{\varepsilon_2} & 0 & 0 & 0 & 0 \\ 0 & 0 & e^{\gamma_2 d_2} & e^{-\gamma_2 d_2} & e^{\gamma_3 d_2} & e^{-\gamma_3 d_2} & 0 & 0 \\ 0 & 0 & -\frac{\gamma_2}{\varepsilon_2} e^{\gamma_2 d_2} & \frac{\gamma_2}{\varepsilon_2} e^{-\gamma_2 d_2} & \frac{\gamma_3}{\varepsilon_3} e^{\gamma_3 d_2} & -\frac{\gamma_3}{\varepsilon_3} e^{-\gamma_3 d_2} & 0 & 0 \\ 0 & 0 & 0 & 0 & e^{\gamma_3 d_3} & e^{-\gamma_3 d_3} & e^{\gamma_4 d_3} & e^{-\gamma_4 d_3} \\ 0 & 0 & 0 & 0 & -\frac{\gamma_3}{\varepsilon_3} e^{\gamma_3 d_3} & \frac{\gamma_3}{\varepsilon_3} e^{-\gamma_3 d_3} & \frac{\gamma_4}{\varepsilon_4} e^{\gamma_4 d_3} & -\frac{\gamma_4}{\varepsilon_4} e^{-\gamma_4 d_3} \\ 0 & 0 & 0 & 0 & 0 & 0 & 1 & 0 \end{pmatrix} = 0 \quad \text{(S3)}$$

where d_n is the thickness of the nth layer. Designating silicon as 1st layer, MXene as 2nd layer, silicon dioxide as 3rd layer, and gold as 4th layer, we analytically obtained the dispersion relation of the AMP mode. To validate our calculation results, we additionally solved the dispersion relation by employing the FDE solver. For the calculations, silicon layer thickness of 1,000 nm, gold layer thickness of 1,000 nm, silicon dioxide layer thickness of 10 nm, and MXene layer thickness of 10, 20, and 80 nm are used. **Figure S2** shows a comparison of the dispersion relation of the AMP mode between analytical calculation and numerical calculation obtained with the FDE solver. As described in **Figure S2**, good agreement between the theoretical calculation and simulation results is observed.

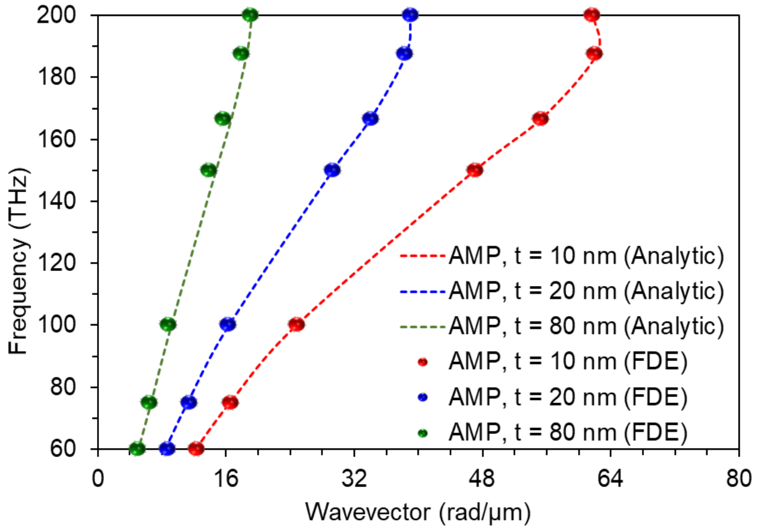

Figure S2. Dispersion relations of AMP mode with MXene thickness of 10 nm (red curve and dot), 20 nm (blue curve and dot), and 80 nm (green curve and dot), respectively. Dotted lines and dots show analytical calculations and numerical calculations, respectively.

Supplementary note 3. Validation of thickness-dependent plasmon confinement using PMMA

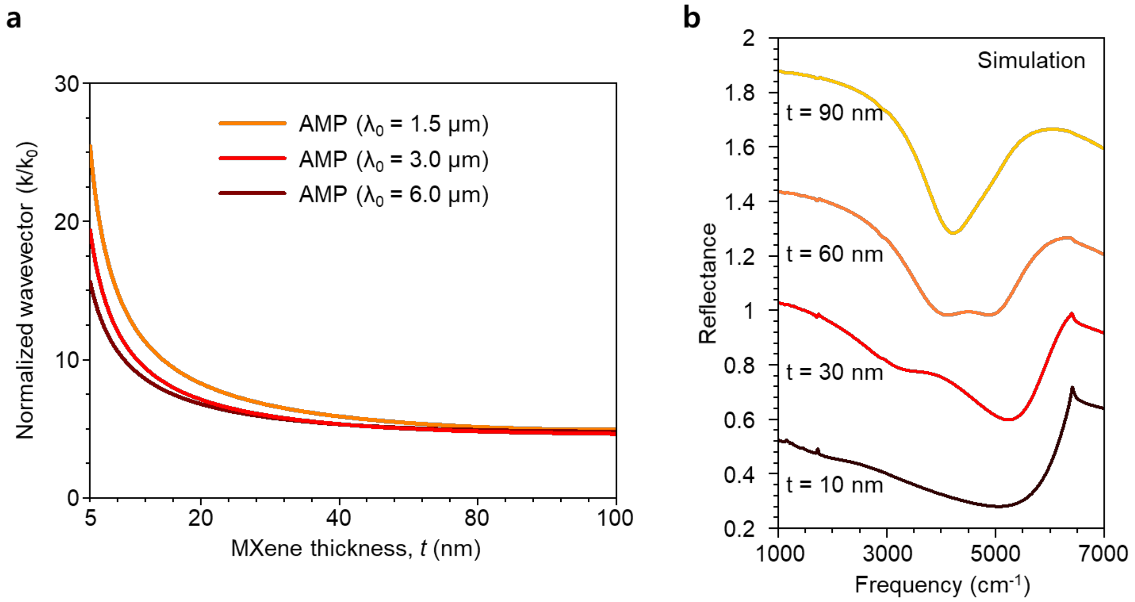

Figure S3. Verification of the MXene-thickness–dependent plasmon confinement using a PMMA analyte layer. a Calculated normalized wavevector (k/k_0) as a function of MXene thickness *t*. **b** Simulated reflectance spectra of the AMP resonator with a PMMA analyte layer for different MXene thicknesses.

Supplementary note 4. Fabrication and SEM characterization of AMP resonators

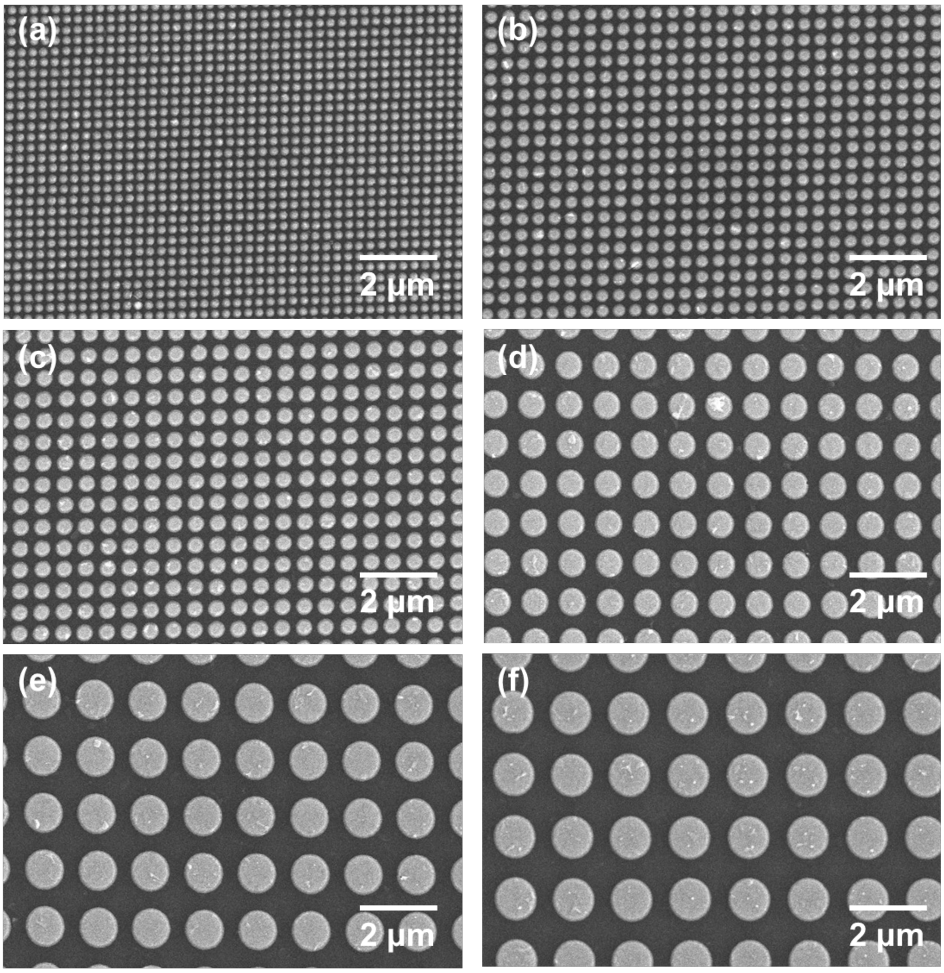

Figure S4. SEM image of fabricated AMP resonator with disk diameters D of (a-f) 260, 360, 470, 790, 1100, and 1200 nm, and corresponding periodicities L of 450, 600, 750, 1200, 1500, and 1800 nm, respectively.

Supplementary note 5. Plasmonic response and field confinement in AMP resonators

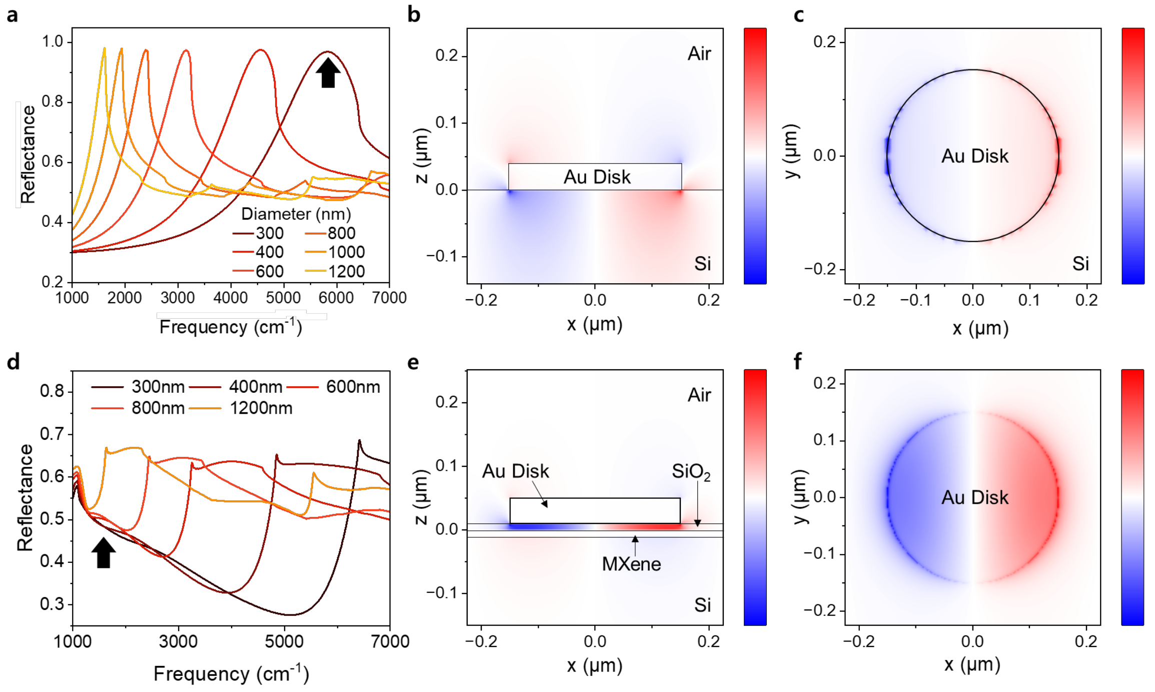

Figure S5. Comparison of plasmonic responses between a bare Au disk resonator and the AMP resonator. (a–c) Bare Au resonator: **(a)** simulated diameter-dependent reflectance spectra. **(b,c)** Simulated electric-field distributions (|E|) at the fundamental resonance (black arrow) in the xz and xy planes, respectively. **(d–f)** AMP resonator (MXene thickness $t = 10$ nm): **(d)** simulated diameter-dependent reflectance spectra. **(e,f)** Corresponding electric-field distributions at the fundamental resonance in the xz and xy planes.

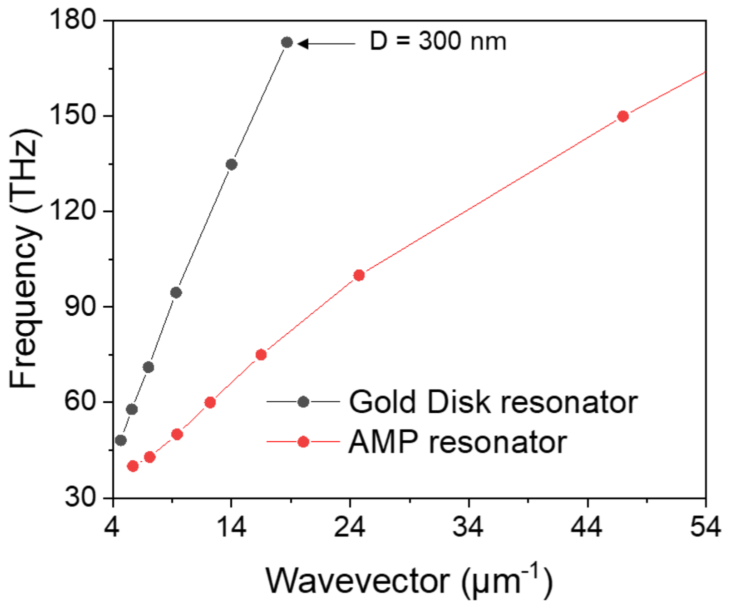

Figure S6. Dispersion relations of the bare Au disk and the AMP resonator.

Supplementary note 6. Thickness control of ultrathin PMMA and GO films

To deposit thin layers of PMMA and GO with nanometer-scale thickness, different spin-coating conditions were employed. The thicknesses of both PMMA and GO films were measured using atomic force microscopy (AFM), as shown in **Figure S7**. For the PMMA thin film shown in **Figure S7a**, a 495 PMMA C2 solution was diluted with monochlorobenzene, and the film thickness was controlled by adjusting the spin speed and its concentration. The spin coating at 3600 rpm with PMMA concentrations of 0.4%, 0.8% and 1.5% yielded film thicknesses of approximately 8, 20, and 40 nm, respectively. For the GO film shown in **Figure S7b**, a commercial solution (HGO6-S30) was diluted to 1% concentration and spin-coated at 6000, 4000, and 2000 rpm to achieve film thicknesses of approximately 10, 20, and 40 nm, respectively.

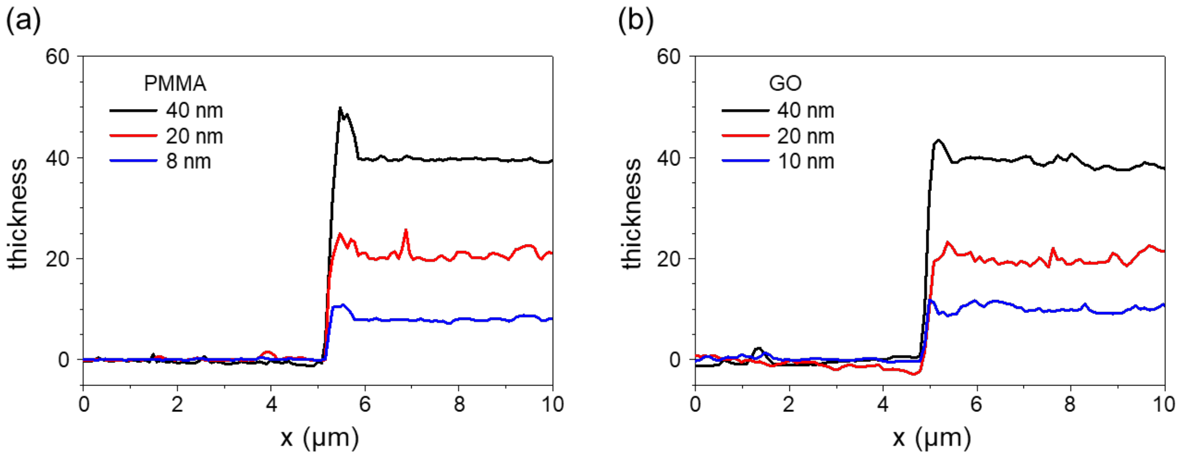

Figure S7. AFM-measured thickness profiles of (a) PMMA and (b) GO thin films with varying spin-coating conditions.

Supplementary note 7. Comprehensive SEIRA spectra and fitting analysis

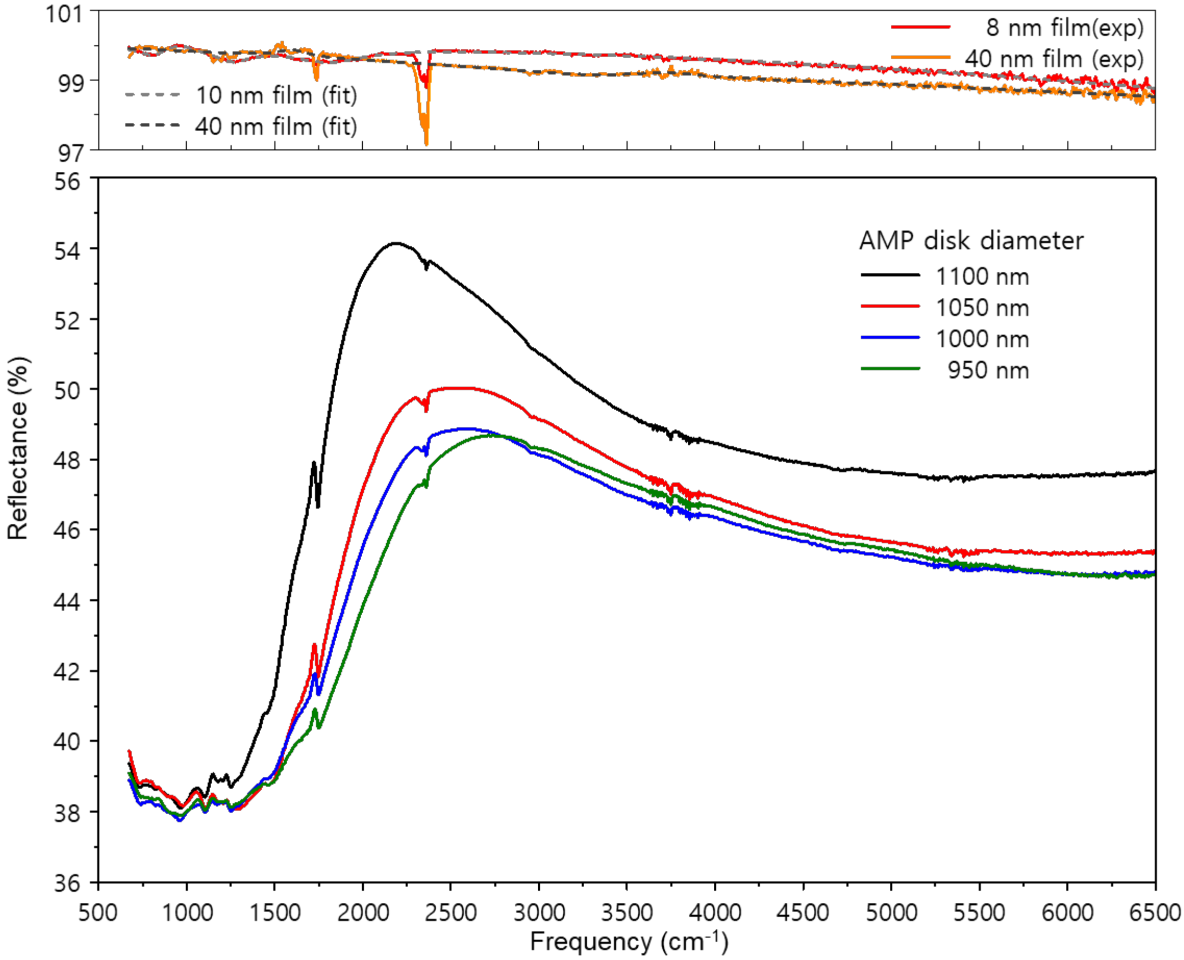

Figure S8. Entire reflectance spectra including both film reference and AMP device measurements for PMMA. The top panel shows the reference spectra of 8 nm and 40 nm PMMA thin films without AMP structures to identify molecular vibrational signatures; solid lines represent measured data and dashed lines represent fitted curves. The bottom panel shows the reflectance spectra of AMP resonators fabricated with an 8 nm PMMA film and varying Au disk diameters (D = 950, 1000, 1050, and 1100 nm) at a fixed period of 1500 nm. The absorption dips near 2350 cm^{-1} and 3750 cm^{-1} originate from ambient CO_2 and H_2O and are not related to the SEIRA AMP device response.

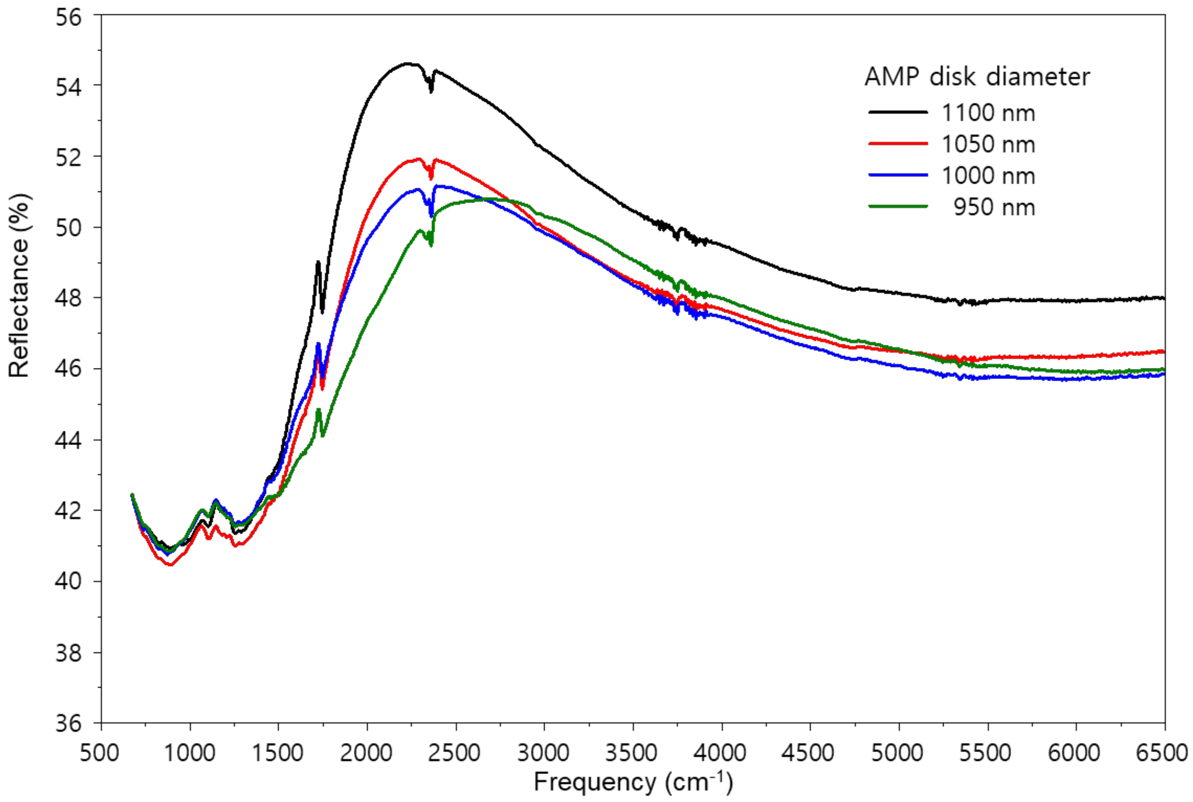

Figure S9. Entire reflectance spectra measured 8-months after fabrication to evaluate long-term chemical stability, including both film reference and AMP device measurements for PMMA.The top panel shows the reference spectra of 8 nm and 40 nm PMMA thin films without AMP structures to identify molecular vibrational signatures; solid lines represent measured data and dashed lines represent fitted curves. The bottom panel shows the reflectance spectra of AMP resonators fabricated with an 8 nm PMMA film and varying Au disk diameters (D = 950, 1000, 1050, and 1100 nm) at a fixed period of 1500 nm. The absorption dips near 2350 cm^{-1} and 3750 cm^{-1} originate from ambient CO_2 and H_2O and are not related to the SEIRA AMP device response.

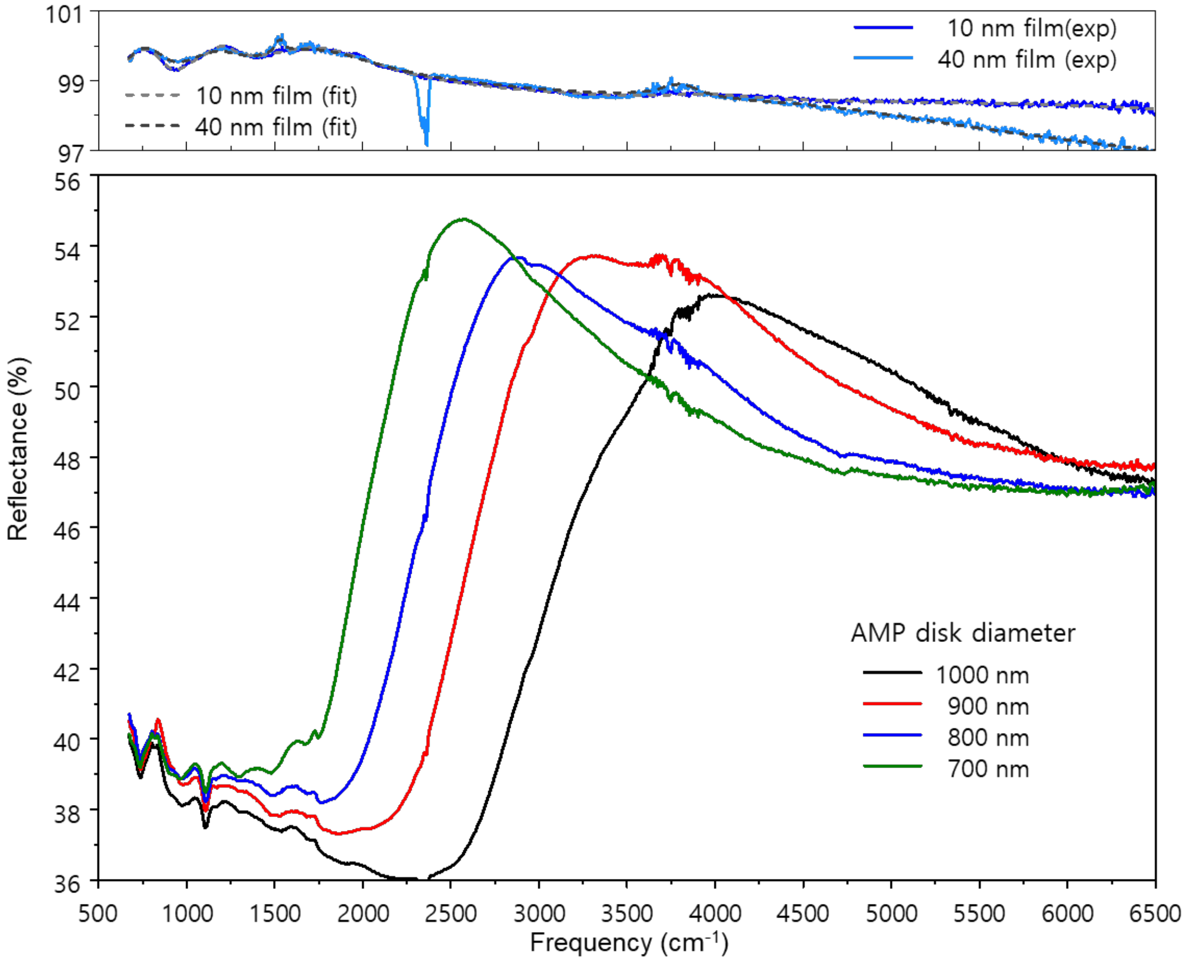

Figure S10. Entire reflectance spectra including both film reference and AMP device measurements for GO. The top panel shows the reference spectra of 10 nm and 40 nm GO films without AMP structures to identify molecular vibrational signatures; solid lines represent measured data and dashed lines represent fitted curves. The bottom panel shows the reflectance spectra of AMP resonators fabricated with an 8 nm GO film and varying Au disk diameters (D = 700, 800, 900, and 1000 nm) with corresponding periods of 1050, 1200, 1350, and 1500 nm. The absorptions near 2350 cm^{-1} and 3750 cm^{-1} originate from ambient CO_2 and H_2O and are not related to the SEIRA AMP device response.

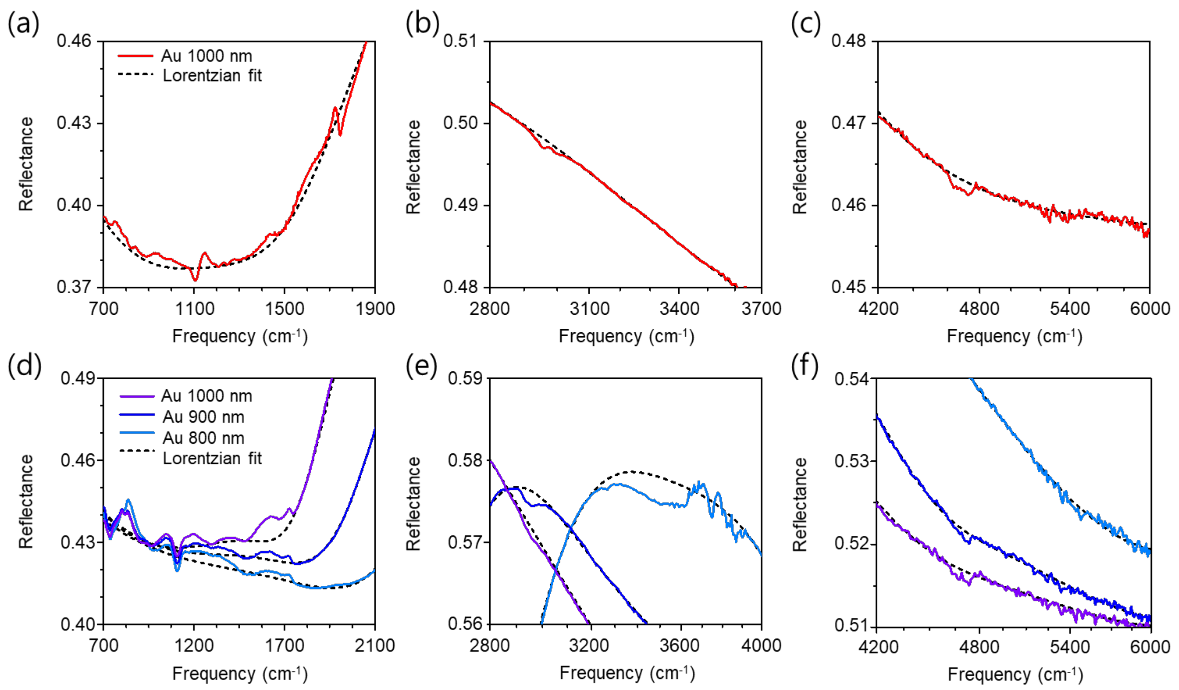

Figure S11. Measured reflectance spectra of AMP resonators with ultrathin analyte films and corresponding Lorentzian fitting curves. (a–c) Reflectance spectra of an AMP resonator with an 8 nm thick PMMA film (solid red lines) and Lorentzian fits (dashed lines), shown for three spectral regions: 700–1900 cm^{-1} (a), 2800–3700 cm^{-1} (b), and 4200–6000 cm^{-1} (c). (d–f) Reflectance spectra of AMP resonators with a 10 nm thick GO film and Lorentzian fitting curves in the regions: 700–2100 cm^{-1} (d), 2800–4000 cm^{-1} (e), and 4200–6000 cm^{-1} (f). GO films were measured using AMP resonators with disk diameters of 1000 nm (magenta), 900 nm (blue), and 800 nm (cyan), respectively.

Supplementary note 8. Comparison of SEIRA responses between AMP and bare Au resonators

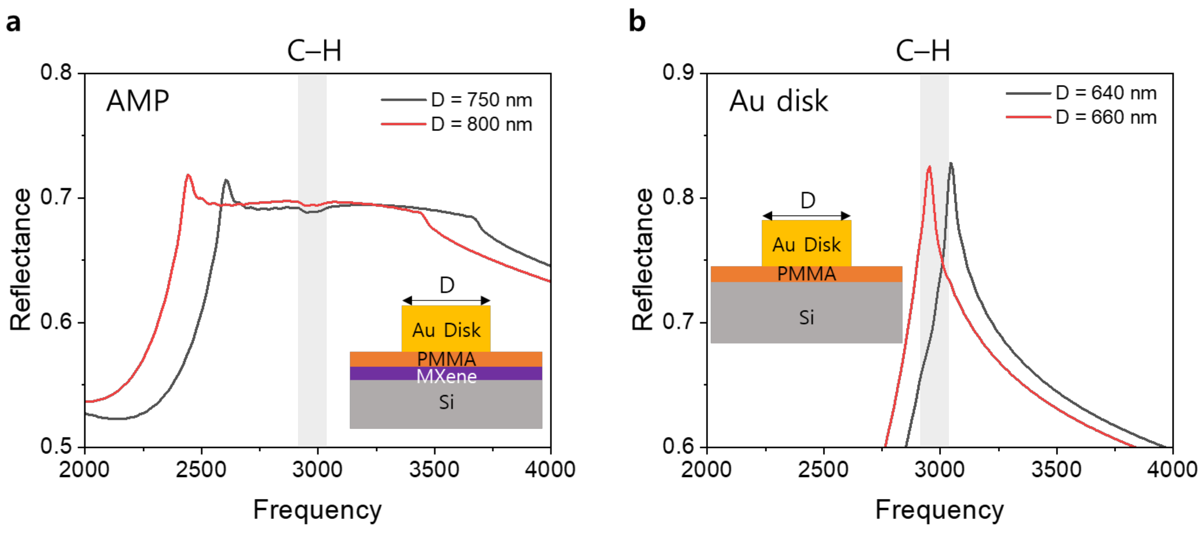

Figure S12. Simulated reflectance spectra targeting the C-H band (~2930 cm^{-1}) for (**a**) the AMP resonator and (**b**) bare Au disk resonator.

Supplementary note 9. XPS characterization of graphene oxide (GO) film

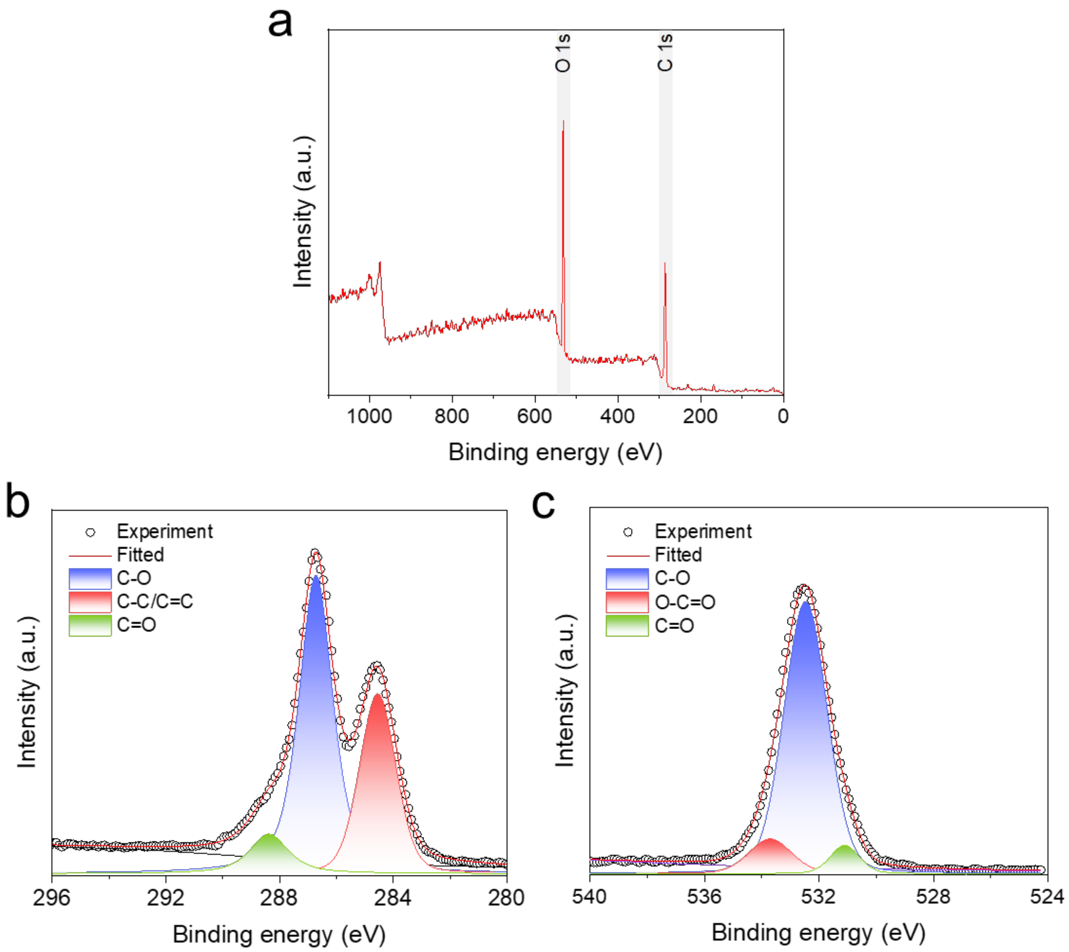

Figure S13. XPS spectra of GO film. **A)** Survey scan and **b-c)** high-resolution core level XPS spectra of **b)** O 1s, and **c)** C 1sO of GO film.
